# Editorial: Automated data curation and data governance automation

**DOI:** 10.3389/fdata.2023.1148331

**Published:** 2023-03-29

**Authors:** John R. Talburt, Lisa Ehrlinger, Justin Magruder

**Affiliations:** ^1^Department of Information Science, University of Arkansas at Little Rock, Little Rock, AR, United States; ^2^Software Competence Center Hagenberg GmbH, Hagenberg, Austria; ^3^Science Applications International Corporation, Reston, VA, United States

**Keywords:** data curation automation, data governance automation, automated error detection, automated error correction, automated data integration

The goal of digital transformation is to create value from data through analytics and machine learning. Over the last decade, many leaders have realized that the full value of their organization's data cannot be realized without effective data governance and management processes, and that technology is but one of the means to that end. Organizations and people are often not aware how and where to find the right data and how to curate it such that its quality is fit for a specific analytics use case. Recently, researchers in artificial intelligence have begun to recognize and address the importance of data curation and data governance under the umbrella term, “data-centric AI.^1^” Some researchers find the terms data curation and data governance unfamiliar, but they represent the foundation of the digital transformation in many organizations around the globe. Despite their criticality, both data curation and data governance require extensive human activity and therefore suffer from a lack of automation. As a result, these critical processes are becoming the bottlenecks of the digital revolution, where data is generated automatically (e.g., by sensors) at ever-increasing rates.

Data and information have a life cycle like that of software development. Just as new software systems are conceived, developed, tested, deployed, and eventually replaced, so are data and information. Data curation is simply the management of data and information through their life cycle from creation, acquisition, assessment, preparation, storage, protection, application, and final disposition. While data curation comprises the rules and decisions made at each stage of the data life cycle, data governance includes the framework, standards, roles, and responsibilities for making these decisions. Data curation has been a practice since computing began, but data governance has emerged recently as the requisite method to bring discipline to data management. Organizations implement data governance to understand what data they have, what it represents, who needs it, where to find it, what it is being used for, and how to ensure it is being used properly.

The problem reminds many of the cobbler's children with no shoes. While there has been extensive research on advanced analytics and machine learning, little has been focused on the automation of data governance and curation. For decades, data scientists and researchers have spent 80% of their efforts finding and preparing data, and only 20% on actual analytics,^2^ to the frustration of virtually everyone involved.

So, what is our vision of the future state? How can we reverse the “Data Scientist Productivity Ratio” and enable 80% of efforts to be devoted to analytics?

For data curation, imagine a self-service model. For example, when Mr. Spock interacts with the computer on the Starship Enterprise, he simply gives a command like “plot a course to the Orion nebula.” The computer understands the command, finds and marshals the data, and makes the calculations. While “self-service analytics” is gaining in popularity, it is typically limited to business users of carefully curated datasets and with limited, “canned” operations. Imagine a future when all users, from developers to data scientists to business users, can find and use all data they require.

Another analogy for the future state of data curation is the Data Washing machine concept (Talburt et al., 2020). We could put all our dirty laundry into a washing machine with some soap, set a few dials, and our clothes come out clean. In the same way, we should be able to put all our datasets into a data washing machine with some metadata and curation rules, and collect our data error free and fully integrated.

The papers in this special issue are moving us forward on several of these fronts toward the vision of automated data curation and data governance. Specifically, the papers contribute as follows:

Ehrlinger and Wöß provide a systematic overview on data curation tools with a special focus on automation capabilities. The findings enable data leaders in selecting the most appropriate tool for a given use case. Only four out of the 13 investigated tools provided full support for the automated execution of data quality rules. The authors conclude that dedicated methods to address data source heterogeneity and alternatives to the manual creation of data quality rules are still open research questions.

Tudoreanu proposes such a novel approach to the automation of data quality curation. He specifically addresses the heterogeneity of data with a distance function that transforms each record to a comparable n-dimensional feature vector. The algorithm allows to deploy data curation methods on real-world settings, where different types of data from structured to semi-structured sources should be analyzed.

AbuHalimeh investigates the impact of poor data in clinical research informatics, a high-stakes domain where quality assurance of analytics is essential. The author specifically addresses the need to make software tools in this context more data quality aware. The paper concludes that designing software under the assumption that data are perfect is no longer acceptable.

The paper by Greer et al. is also dedicated to the quality-critical health domain. In addition to the measurement of data quality, the authors specifically address the improvement of data quality with an approach to enrich data with so-called “social determinants of health” (SDOH). The research shows that the enrichment (consisting of mapping, linking, quality analysis, preprocessing, and storage) of SDOH enables clinicians to improve patient treatment and care.

Finally, Pierce completes the message of this special issue by arguing for the parity between processing efficiency and data performance, the primary goal of digital transformation. Pierce proposes a balanced scorecard approach that supports organizations in designing meaningful metrics for maximizing the potential of their data assets. The paper also discusses implementation challenges of data governance strategies and emphasizes the importance of the Chief Data Officer role, the person leading an organization's data strategy.

## Author contributions

All authors listed have made a substantial, direct, and intellectual contribution to the work and approved it for publication.

## Footnotes

^1^ https://datacentricai.org

^2^Cleaning Data: Most Time-Consuming, Least Enjoyable Data Science Task, Gil Press, Forbes. http://www.forbes.com/sites/gilpress/2016/03/23/data-preparation-most-time-consuming-least-enjoyable-data-science-task-survey-says.
